# Erythropoietin as a critical prognostic indicator in ICU patients with sepsis: a prospective observational study

**DOI:** 10.1186/s40560-025-00787-x

**Published:** 2025-03-20

**Authors:** Qianping Zhang, Yan Zhang, Xinyi Tian, Kaifan Lin, Jie Weng, Xinyi Fu, Yongjie Chen, Xuemeng Li, Bihuan Cheng, Xiaolong Zhang, Yuqiang Gong, Shengwei Jin, Ye Gao

**Affiliations:** 1 Department of Anesthesia, Pain and Critical Care, The Second Affiliated Hospital and Yuying Children’s Hospital of Wenzhou Medical University, Wenzhou, Zhejiang China; 2 NHC Key Lab of Reproduction Regulation, Shanghai Engineering Research Center of Reproductive Health Drug and Devices, Shanghai Institute for Biomedical and Pharmaceutical Technologies, Shanghai, 200237 China; 3 The Second Clinical Medical College of Wenzhou Medical University, Wenzhou, Zhejiang China

**Keywords:** Erythropoietin, Sepsis, 28-day mortality

## Abstract

**Background:**

Erythropoietin (EPO), a glycoprotein hormone primarily produced in the kidneys, plays pleiotropic roles in hematopoietic and non-hematopoietic system. However, the clinical relevance of circulating EPO in sepsis progression and outcomes remains contentious and requires further elucidation.

**Methods:**

Participants were categorized into three groups on the basis of EPO tertiles. The primary outcome was 28-day mortality. Multivariate Cox proportional regression analysis and restricted cubic spline regression were employed to evaluate the association between EPO levels and 28-day mortality in sepsis patients. Subgroup analyses were also conducted. Causal mediation analysis was conducted to explore the potential mediating role of EPO in the relationship between lactate and 28-day mortality.

**Results:**

A total of 267 patients (65.17% male) were included in the study. The 28-day and hospital mortality rates were 23.22 and 31.20%, respectively. Multivariate Cox regression revealed significantly higher 28-day and hospital mortality in the highest EPO tertile compared to the lowest (HR 2.93, 95% CI 1.20–7.22; HR 2.47, 95% CI 1.05–5.81, respectively). Restricted cubic spline analysis demonstrated a progressively increasing mortality risk with elevated EPO levels. Subgroup analyses confirmed the consistency and stability of the effect size and direction across different subgroups. Moreover, causal intermediary analysis revealed that the association between lactate and 28-day mortality was partially mediated by EPO, with a mediation ratio of 12.59%.

**Conclusions:**

Elevated EPO levels in patients with sepsis are correlated with unfavorable prognoses and may function as a prognostic biomarker for adverse outcomes.

**Supplementary Information:**

The online version contains supplementary material available at 10.1186/s40560-025-00787-x.

## Background

Sepsis is a life-threatening condition that arises from infection-induced immune dysfunction, leading to subsequent organ failure [1]. Globally, it is estimated that there are 4.89 million cases of sepsis, with sepsis-related deaths totaling 11 million, accounting for 19.7% of the global mortality [2]. This situation imposes a substantial economic burden on healthcare systems and presents an urgent challenge for public health, particularly in low- and middle-income countries [3]. Early identification of critically ill patients with sepsis is crucial for improving sepsis prognosis [4–6]. Although inflammatory markers such as c-reactive protein (CRP) and procalcitonin (PCT) are utilized to predict the severity and prognosis of sepsis, their accuracy and reliability are often the subject of debate [7]. Therefore, there is an urgent need to identify better biomarkers for early recognition of critically ill patients with sepsis.

Erythropoietin (EPO) is a glycoprotein hormone produced primarily in the kidneys that regulates red blood cell production. Its synthesis is stimulated by low oxygen levels. This hypoxia-driven increase in EPO production is intended to enhance oxygen delivery to tissues by promoting erythrocyte maturation and release in the bone marrow [8–11]. Recent studies have highlighted the non-erythropoietic functions of EPO, which include anti-inflammatory, antioxidant, and cytoprotective properties [12–15]. These actions contribute to attenuation of the inflammatory response, preservation of immune homeostasis, and mitigation of the severity and duration of inflammation [16–19]. Several studies have demonstrated that the serum EPO concentration can serve as an early warning indicator of poor prognosis in patients with conditions, such as diabetic chronic kidney disease [20], renal transplantation [21], and heart failure [22].

In the context of sepsis, non-survivors have been observed to exhibit elevated levels of EPO compared with survivors, with endogenous EPO concentrations correlated with indicators of tissue hypoperfusion, such as lactate and the carbon dioxide gap [23]. However, a study has reached the opposite conclusion [24], and the correlation between EPO and the prognosis of patients with sepsis is controversial. Therefore, the aim of this study was to assess the prognostic value of early measurement of the EPO concentration in septic patients admitted to the intensive care unit (ICU).

## Methods

### Study design and population

This prospective observational study enrolled patients with sepsis who were admitted to the ICU of the Second Affiliated Hospital of Wenzhou Medical University between October 1, 2022, and December 30, 2023. The inclusion criteria consisted of: (1) adherence to the sepsis 3.0 diagnostic criteria [1] and (2) completion of various laboratory tests, including EPO, hemoglobin, CRP, PCT, and others, within 48 h of ICU admission. The exclusion criteria included the following: (1) physiological states related to pregnancy and lactation; (2) the presence of malignant neoplasms undergoing chemotherapy or radiotherapy, organ transplantation, hormonal therapies, or immunosuppressive agents; (3) advanced chronic illnesses (e.g., chronic kidney disease at stages 4 and 5, chronic heart failure classified as NYHA III and IV, end-stage cancer, and COPD at GOLD stages 3 and 4) [25–28]; (4) interhospital transfer cases; and (5) receiving exogenous EPO therapy, such as recombinant human erythropoietin injections. The study was conducted in accordance with the principles outlined in the Declaration of Helsinki and received approval from the Medical Ethics Committee of the Second Affiliated Hospital of Wenzhou Medical University (ethics approval number: LCKY2020-36).

### Data collection

Demographic data, including age, sex, and medical history, were collected from the enrolled patients. EPO levels were assessed within 48 h of ICU admission. Additional parameters documented included complete blood count, liver and kidney function tests, CRP, PCT, white blood cell (WBC), arterial oxygen pressure, arterial carbon dioxide pressure, and lactate levels. For data that exceeded the detection limits, the maximum detectable value was utilized. The oxygenation index, age-adjusted Charlson comorbidity index (aCCI), and sequential organ failure assessment (SOFA) score were computed from the collected data. The primary outcome indicator for this study was 28-day mortality, while hospital mortality served as the secondary outcome indicator. Clinical nurses collected blood samples for EPO and other laboratory analyses, which were subsequently sent to the laboratory of the Second Affiliated Hospital of Wenzhou Medical University for processing.

### Statistical analysis

Statistical analyses were performed via R software (version 4.3.1) and SPSS software (version 26). The normality of continuous variables was assessed via the Kolmogorov‒Smirnov (K‒S) test. Normally distributed data are presented as the mean ± standard deviation (X ± S), with comparisons between two groups conducted via the independent Student’s *t* test, whereas comparisons among multiple groups were performed via ANOVA (*F* test). For data that did not follow a normal distribution, the results are reported as the median (interquartile range) [median (P25, P75)]. Comparisons between two groups were analyzed with the Mann‒Whitney *U* test, and differences among multiple groups were evaluated via the Kruskal‒Wallis rank‒sum test. Categorical variables are expressed as numbers and percentages (%), with comparisons performed via the chi-square test or Fisher’s exact test as appropriate. Spearman correlation analysis was employed to assess the relationships between continuous variables. Kaplan‒Meier survival curves were generated to illustrate the cumulative survival rate of patients with sepsis, with differences analyzed via the log-rank test. Cox proportional hazards models were used to calculate the hazard ratio (HR) and 95% confidence interval (CI) for the relationship between EPO and the endpoints, adjusting for clinical expertise and prior literature. Variance inflation factors (VIFs) were assessed to detect multicollinearity among variables, where a VIF < 10 suggested that multicollinearity was unlikely to affect estimation.

The specific variables adjusted for are as follows. Model 1: No variables were adjusted. Model 2: Adjusted for age, gender, hypertension, and diabetes. Model 3: In addition to the variables in Model 2, we further adjusted for SOFA score, hemoglobin levels, platelet count (PLT), PCT, glucose, and albumin. Owing to the skewed distribution of EPO, data were transformed using natural logarithms prior to analysis and treated as continuous variables (per 1-SD increment) in multivariate models and mediation analyses. Furthermore, the nonlinear associations between baseline EPO and 28-day, ICU, and in-hospital mortality were explored via a restricted cubic spline regression model with four knots.

The performance of EPO, lactate, SOFA, lactate + SOFA, and EPO + lactate + SOFA was evaluated by assessing their discriminative ability through receiver operating characteristic (ROC) curves and calculating the area under the ROC curve (AUC). In addition, the net reclassification index (NRI) and the integrated discrimination improvement (IDI) index were utilized to further examine the incremental predictive value of EPO + lactate + SOFA in comparison with EPO alone. Stratified analyses were conducted on the basis of age, sex, the SOFA score, lactate levels, oxygenation index, hypertension, diabetes, CKD and types of infections to determine the consistency of the prognostic value of the EPO for outcomes. All the statistical tests were two-tailed, with a significance level established at *P* < 0.05.

## Results

### Baseline characteristics

**Fig. 1:**
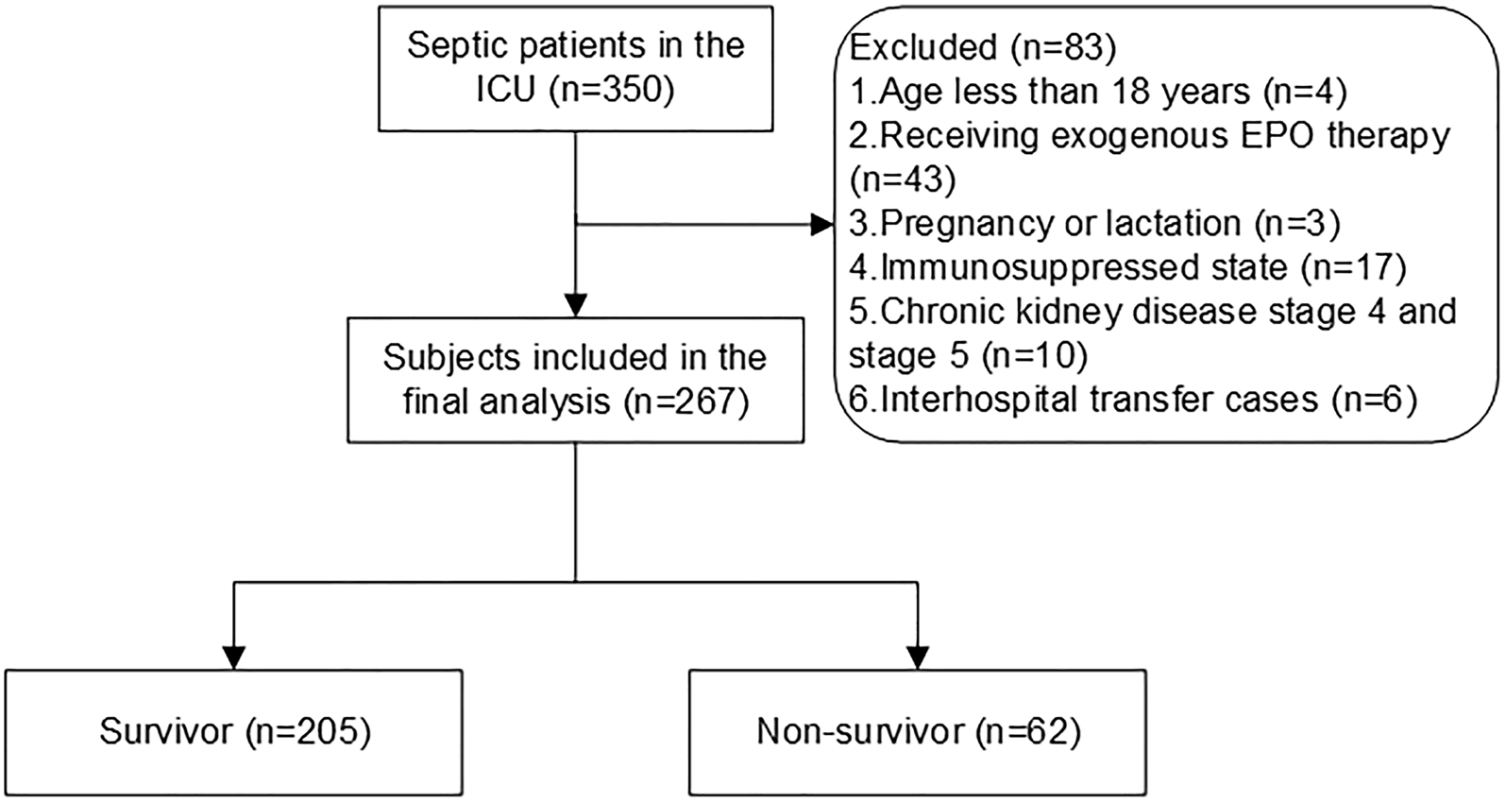
Flowchart of the study cohort

In this prospective cohort study, 267 out of the 350 subjects with sepsis met the inclusion criteria (Fig. 1). The in-hospital and 28-day mortality rates were 32.20% and 23.22%, respectively. Table 1 presents a comparison between patients in the EPO-T1 group and those in the other groups. Notably, patients with elevated EPO levels presented lower hemoglobin levels, red blood cell count, and platelet counts, and a greater incidence of septic shock. Furthermore, these patients had increased SOFA scores, lactate levels, ALT, total bilirubin, and potassium concentrations. The group with elevated EPO levels also experienced higher in-hospital and 28-day mortality rates. In addition, the differences in baseline characteristics between survivors and non-survivors during their hospital stay are shown in Table 2.

**Table 1:** Characteristics and outcomes of participants stratified by EPO status

Categories	T1 (*N* = 89)	T2 (*N* = 89)	T3 (*N* = 89)	*P* value
Age (years)	72.00 (55.00–79.00)	68.00 (56.00–74.00)	63.00 (53.00–77.00)	0.222
Gender (male)	60 (67.42%)	56 (62.92%)	58 (65.17%)	0.820
Comorbidities				
Stroke	31 (34.83%)	26 (29.21%)	18 (20.22%)	0.091
Heart disease	16 (17.98%)	11 (12.50%)	13 (14.61%)	0.589
Lung disease	8 (8.99%)	3 (3.37%)	7 (7.87%)	0.286
Liver disease	10 (11.36%)	9 (10.11%)	15 (16.85%)	0.359
Malignant tumor	13 (14.61%)	14 (15.73%)	23 (25.84%)	0.107
CKD	15 (16.85%)	7 (7.87%)	13 (14.61%)	0.181
CKD classification				0.370
1	3 (3.37%)	4 (4.49%)	3 (3.37%)	
2	6 (6.74%)	2 (2.25%)	4 (4.49%)	
3	6 (6.74%)	1 (1.12%)	6 (6.74%)	
Hypertension	57 (64.04%)	40 (44.94%)	29 (32.58%)	<0.001
Diabetes	27 (30.34%)	20 (22.47%)	23 (25.84%)	0.489
Septic shock	9 (10.11%)	20 (22.47%)	38 (42.70%)	<0.001
Types of infections				0.918
Respiratory infections	61 (68.54%)	57 (64.04%)	58 (65.17%)	
Abdominal infections	16 (17.98%)	14 (15.73%)	15 (16.85%)	
Urinary tract infections	6 (6.74%)	8 (8.99%)	9 (10.11%)	
Other infections	6 (6.74%)	10 (11.24%)	7 (7.87%)	
aCCI	6.00 (4.00–7.00)	5.00 (4.00–7.00)	6.00 (3.00–8.00)	0.375
SOFA	6.00 (4.00–9.00)	6.00 (4.00–9.00)	9.00 (6.00–13.00)	<0.001
EPO (mIU/ml)	10.53 (5.88–12.77)	28.07 (21.96–35.18)	128.34 (79.96–371.43)	<0.001
Hemoglobin (g/dL)	115.30 ± 21.47	105.18 ± 19.82	90.83 ± 21.91	<0.001
WBC (*10^9^/L)	10.15 (8.26–13.15)	12.30 (9.07–15.67)	11.29 (8.68–16.99)	0.074
RBC	3.83 ± 0.80	3.43 ± 0.65	3.04 ± 0.78	<0.001
MCV (fL)	92.00 (89.90–94.80)	93.10 (89.20–95.90)	91.00 (87.10–95.50)	0.136
RDW (%)	13.30 (12.80–14.30)	13.60 (12.60–14.60)	14.20 (13.10–15.80)	0.001
CRP (mg/L)	108.32 (53.10–196.13)	101.52 (50.24–177.11)	106.22 (45.92–170.00)	0.777
PCT (ng/ml)	1.53 (0.34–6.35)	1.06 (0.28–8.31)	3.44 (0.47–13.78)	0.066
Lactate (mmol/L)	1.40 (1.10–1.70)	1.40 (1.10–2.00)	2.00 (1.28–3.60)	<0.001
PH	7.40 (7.36–7.45)	7.42 (7.38–7.44)	7.41 (7.36–7.45)	0.612
Oxygenation index (mmHg)	241.52 (176.89–293.03)	260.00 (193.00–321.21)	239.67 (153.20–320.61)	0.567
Glucose (mmol/L)	7.53 (6.17–10.71)	7.12 (5.85–8.61)	7.71 (6.44–9.65)	0.387
ALT	21.00 (14.00–53.00)	21.00 (14.00–41.00)	41.00 (18.00–97.00)	0.002
Albumin (g/L)	30.27 ± 3.64	30.03 ± 4.46	29.23 ± 4.23	0.210
Total bilirubin	16.10 (10.10–23.90)	15.40 (11.30–21.80)	22.00 (12.80–49.30)	0.002
Creatinine (μmol/L)	84.50 (64.00–142.70)	82.30 (56.40–120.00)	93.50 (67.90–202.80)	0.067
Sodium (mmol/L)	141.50 (138.70–144.30)	141.00 (138.70–144.20)	143.00 (139.00–146.40)	0.185
Potassium (mmol/L)	3.80 (3.58–4.13)	3.73 (3.57–4.10)	4.05 (3.59–4.48)	0.009
Phosphorus (mmol/L)	0.94 (0.72–1.14)	0.86 (0.66–1.13)	0.98 (0.65–1.42)	0.215
Event				
Hospital-mortality	19 (21.59%)	27 (30.34%)	37 (41.57%)	0.016
28-Day mortality	8 (8.99%)	17 (19.10%)	37 (41.57%)	<0.001
LOS ICU days(days)	6.00 (3.25–12.00)	7.00 (3.00–16.00)	6.00 (2.75–13.00)	0.599
LOS hospital, days (days)	21.00 (13.00–33.00)	18.00 (12.00–37.00)	19.00 (9.00–30.00)	0.181
ICU free days(days)	22.00 (13.00–24.00)	19.00 (0.00–25.00)	10.00 (0.00–22.00)	<0.001
Hospital free days(days)	3.00 (0.00–12.00)	0.00 (0.00–13.00)	0.00 (0.00–4.00)	0.002

Data: *N* (%) or mean (Q1–Q3) or mean ± standard deviation

*aCCI* age-adjusted charlson comorbidity index, *WBC* white blood cell count, *MCV* mean cell volume, *RDW* red cell distribution width, *CRP* c-reactive protein, *PCT* procalcitonin, *BUN* blood urea nitrogen, *ALT* alanine aminotransferase, *SOFA* sequential organ failure assessment, *LOS ICU days* intensive care unit length of stay, *LOS hospital day* hospital length of stay, *CKD* chronic kidney disease

**Table 2:** Baseline characteristics of the Survivor and Non-survivor groups

Categories	Overall (*n* = 267)	Survivor (*n* = 205)	Non-survivor (*n* = 62)	*P* value
Age (years)	68.00 (54.00–77.00)	67.00 (53.00–76.00)	69.00 (58.25–79.00)	0.304
Gender (male)	174 (65.17%)	130 (63.41%)	44 (70.97%)	0.274
Comorbidities				
stroke	75 (28.08%)	56 (27.32%)	19 (30.65%)	0.609
Heart disease	40 (14.98%)	27(13.24%)	13 (20.97%)	0.136
Lung disease	18 (6.74%)	14 (6.83%)	4 (6.45%)	0.917
Liver disease	34 (12.73%)	26 (12.75%)	8 (12.90%)	0.974
Malignant tumor	50 (18.73%)	36 (17.56%)	14 (22.58%)	0.375
CKD	35 (13.11%)	26 (12.68%)	9 (14.52%)	0.708
CKD classification				0.438
1	10 (3.75%)	9 (4.40%)	1 (1.61%)	
2	12 (4.49%)	9 (4.39%)	3 (4.84%)	
3	13 (4.69%)	8 (3.90%)	5 (8.06%)	
Hypertension	126 (47.19%)	97 (47.32%)	29 (46.77%)	0.940
Diabetes	70 (26.22%)	53 (25.85%)	17 (27.42%)	0.806
Septic shock	67 (25.09%)	35 (17.07%)	32 (51.61%)	<0.001
Types of infections				0.411
Respiratory infections	176 (65.92%)	130 (63.41%)	46 (74.19%)	
Abdominal infections	45(16.85%)	36 (17.56%)	9 (14.52%)	
Urinary tract infections	23 (8.61%)	20 (9.76%)	3 (4.84%)	
Other infections	23 (8.61%)	19 (9.27%)	4 (6.45%)	
aCCI	6.00 (4.00–7.00)	5.38 (3.00–7.00)	6.27 (4.25–8.00)	0.032
SOFA score	7.00 (5.00–10.00)	6.00 (4.00–9.00)	11.00 (7.00–13.00)	<0.001
Laboratory tests				
EPO (mIU/ml)	29.23 (12.64–93.60)	22.81(11.29–51.66)	75.25 (30.10–447.19)	<0.001
Hemoglobin (g/L)	102.92 ± 23.59	105.42 ± 22.32	98.32 ± 25.68	0.035
WBC(*10^9^/L)	11.21 (8.44–15.86)	11.07 (8.34–14.37)	12.46 (8.70–19.49)	0.067
CRP (mg/L)	105.06 (48.41–182.49)	108.75 (52.18–185.35)	99.11(42.31–172.67)	0.369
PCT (ng/ml)	1.92 (0.43–10.59)	1.50 (0.42–9.21)	3.90 (0.41–13.13)	0.377
Lactate (mmol/L)	1.50 (1.10–2.30)	1.40 (1.10–1.90)	2.50 (1.50–6.10)	<0.001
Oxygenation index (mmHg)	255.72 ± 116.05	254.00 (190.61–320.00)	225.33 (124.44–293.45)	0.036
Albumin (g/L)	29.82 ± 4.11	30.06 ± 3.94	29.13 ± 4.68	0.123
Creatinine (μmol/L)	87.55 (63.95–151.85)	81.80 (62.60–121.30)	107.00 (68.38–242.82)	0.033
Event				
LOS ICU days	6.00 (3.00–13.00)	6.00 (3.00–14.00)	7.00 (3.00–12.75)	0.593
LOS hospital day	20.00 (12.00–33.00)	24.00 (15.00–38.00)	11.00 (5.25–19.00)	<0.001

Data: *N* (%) or mean (Q1–Q3) or mean ± standard deviation

*aCCI* age-adjusted charlson comorbidity index, *WBC* white blood cell count, *CRP* c-reactive protein, *PCT* procalcitonin, *BUN* blood urea nitrogen, *SOFA* sequential organ failure assessment, *LOS ICU days* intensive care unit length of stay, *LOS hospital day* hospital length of stay, *CKD* chronic kidney disease

### EPO and 28-day mortality, and hospital mortality

**Fig. 2:**
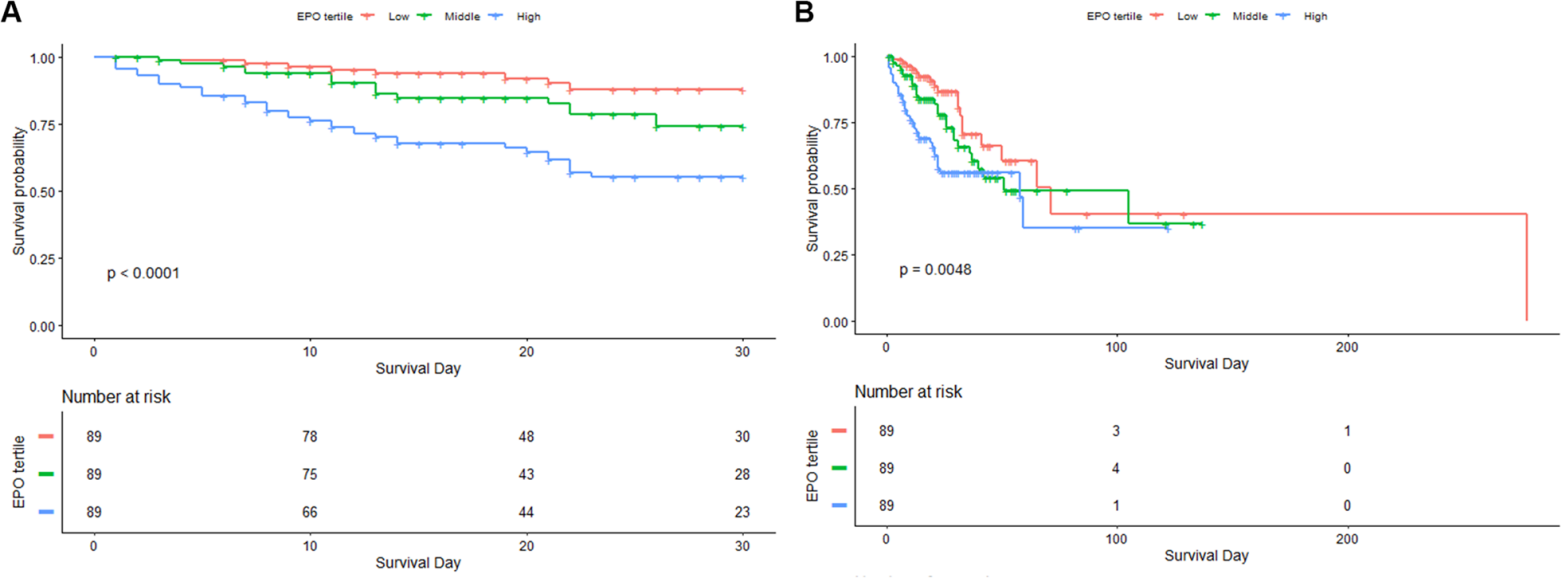
Kaplan–Meier survival analysis curves for all-cause mortality. Kaplan–Meier curves showing cumulative probability of 28 days (**A**), and hospital mortality (**B**)

**Fig. 3:**
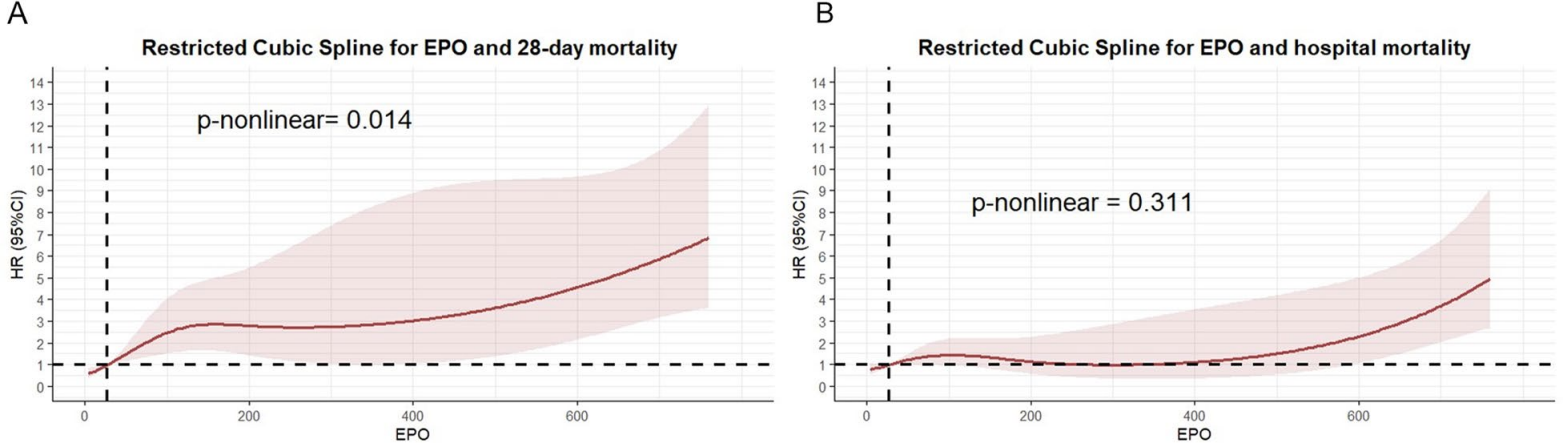
**A** Restricted cubic spline for 28-day mortality. **B** Restricted cubic spline for hospital mortality

Kaplan‒Meier survival analysis curves were generated to assess the incidence of primary outcomes across groups categorized by EPO tertiles (Fig. 2). Patients with elevated EPO levels had a significantly higher risk of 28-day mortality (log-rank *P* < 0.0001) and hospital mortality (log-rank *P* < 0.0048). Cox proportional hazards analysis was conducted to investigate the relationships between EPO levels and both 28-day mortality and hospital mortality. The findings indicated that EPO was independently associated with an increased risk of 28-day mortality (HR 1.3086 [95% CI (1. 0304–1.6618)]; *P* = 0.0274). However, the correlation with hospital mortality did not reach statistical significance (HR 1.1272; [95% CI (0. 9202–1.3808)]; *P* = 0.2473). In the fully adjusted Model 3, the hazard ratio (HR) for 28-day mortality in the highest EPO tertile was 2.9382 (95% CI 1.1963–7.2165), whereas for hospital mortality, it was 2.4692 (95% CI 1.0500–5.8069), both compared with the lowest tertile (Table 3). To ensure the robustness of our findings, a sensitivity analysis was conducted by excluding all CKD patients. The results of this analysis remained consistent with the original findings, thereby confirming the stability of our conclusions. Detailed results are provided in Appendix eTable 1. In addition, the restricted cubic splines regression model indicated that the risk of 28-day mortality increased nonlinearly with increasing EPO levels (*P* for nonlinearity = 0.014). Conversely, the risk of hospital mortality exhibited a linear increase with higher EPO levels (*P* for nonlinearity = 0.311) (Fig. 3A, B).

**Table 3:** Association between EPO and 28-day mortality

Categories	Model 1		Model 2		Model 3	
	HR (95% CI)	*P* value	HR (95% CI)	*P* value	HR (95% CI)	*P* value
28-day mortality						
EPO*	1.7169 (1.4529, 2.0288)	<0.0001	1.7515 (1.4815, 2.0707)	<0.0001	1.3086 (1.0304, 1.6618)	0.0274
EPO tertile						
T1	1.0	1.0	1.0			
T2	2.2111 (0.9542, 5.1237)	0.0642	2.3883 (1.0262, 5.5588)	0.0434	2.3926 (1.0195, 5.6151)	0.0450
T3	5.7190 (2.6620, 12.2865)	0.0001	6.4253 (2.9470, 14.0090)	0.0001	2.9382 (1.1963, 7.2165)	0.0187
Hospital mortality						
EPO*	1.3988 (1.1983, 1.6328)	0.0002	1.4945 (1.2754, 1.7512)	<0.0001	1.1272 (0.9202, 1.3808)	0.2473
EPO tertile						
T1	1.0		1.0		1.0	
T2	1.5407 (0.8483, 2.7981)	0.1557	1.6748 (0.9161, 3.0620)	0.0939	1.7016 (0.7191, 4.0266)	0.2265
T3	2.4341 (1.3847, 4.2789)	0.0020	3.0130 (1.6744, 5.4217)	0.0002	2.4692 (1.0500, 5.8069)	0.0383

Model 1: Unadjusted

Model 2: Adjusted for age, gender, hypertension, and diabetes

Model 3: Adjusted for age, gender, hypertension, diabetes, SOFA score, Hemoglobin, PLT, PCT, glucose, and albumin

^*^EPO was log-transformed before analysis

**Fig. 4:**
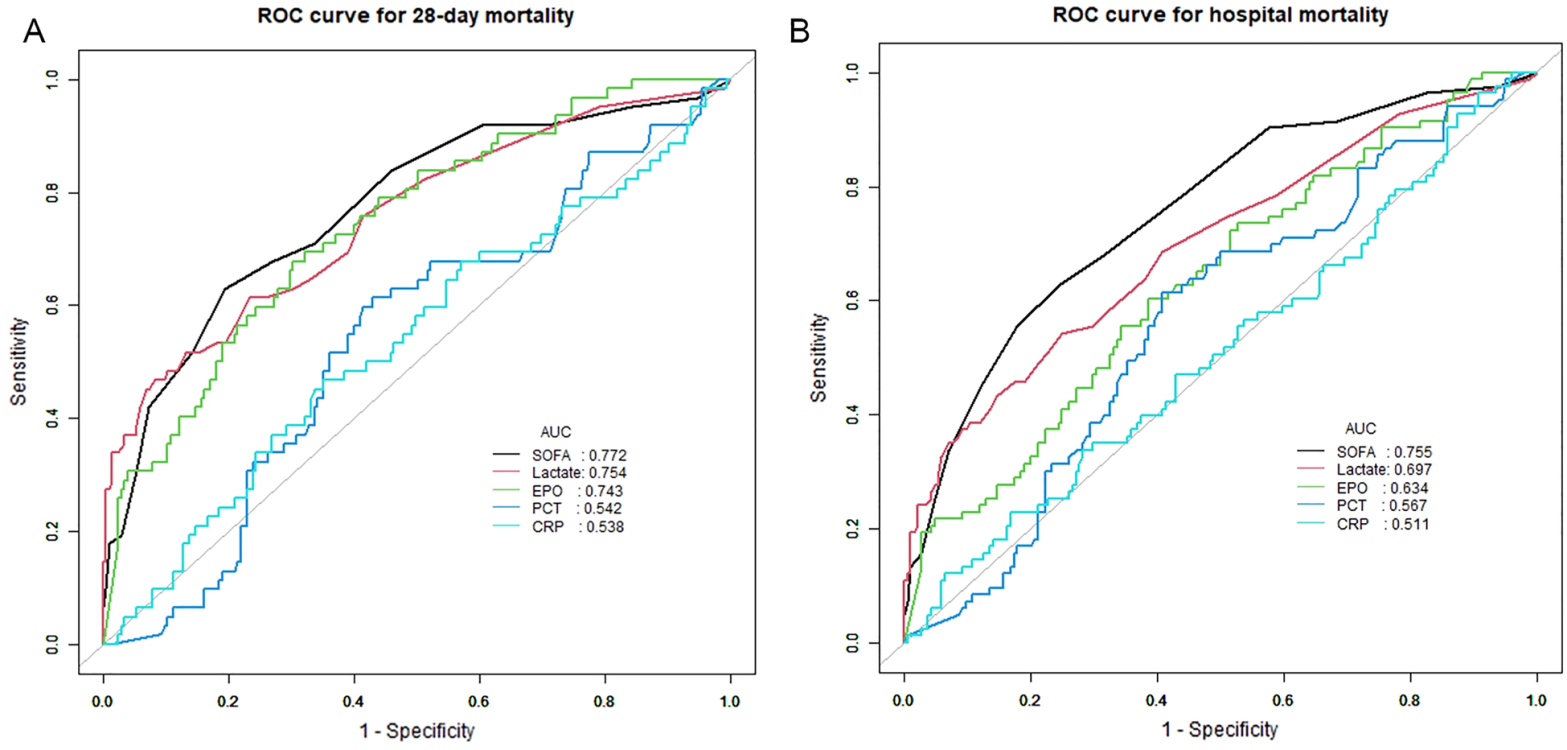
ROC Curves for Evaluating EPO, Lac and SOFA Score of 28 days (**A**), and hospital mortality (**B**)

As shown in Fig. 4, the AUC values for EPO, the SOFA score, and lactate were comparable, measuring 0.743, 0.772, and 0.754, respectively. Notably, the combination of EPO with the SOFA score and lactate levels resulted in increases in both the AUC and specificity. However, the differences among the models did not reach statistical significance, as indicated by the non-significant net reclassification improvement (NRI) and integrated discrimination improvement (IDI) for both 28-day mortality and hospital mortality (all *P* > 0.05) (Table 4).

**Table 4:** NRI and IDI index for EPO combined with lactate and SOFA

	NRI		IDI	
	NRI (95% CI)	*P* value	IDI (95% CI)	*P* value
EPO vs. Lactate	0.0814 (−0.1695, 0.1498)	0.9039	0.0820 (−0.1706, 0.1590)	0.9046
EPO vs. SOFA	0.0724 (−0.049, 0.2350)	0.1992	0.0729 (−0.0498, 0.2358)	0.2018
EPO vs. SOFA + Lactate	0.0781 (−0.0679, 0.2382)	0.2756	0.0786 (−0.0689, 0.2391)	0.2785
EPO vs. EPO + SOFA + Lactate	0.0724 (−0.2350, 0.0490)	0.1992	0.0729 (−0.2358, 0.0498)	0.2018

### Subgroup analysis

**Fig. 5:**
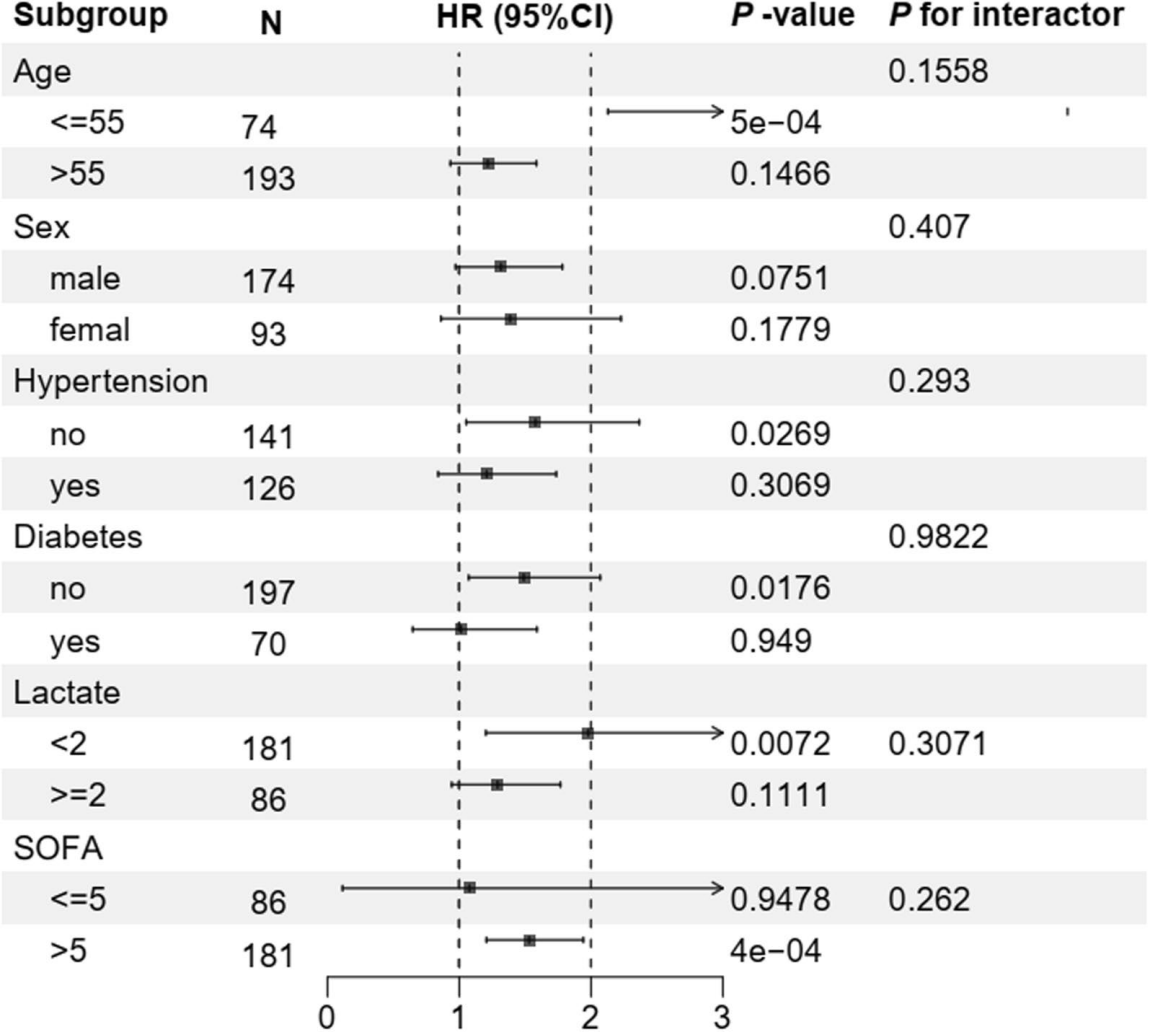
Forest plots of hazard ratios for the hospital mortality in different subgroups

To confirm the relationship between EPO and 28-day mortality, stratified analyses were conducted on the basis of age, sex, the SOFA score, lactate levels, hypertension, diabetes, CKD and types of infections. As illustrated in Fig. 5, EPO was significantly associated with an increased risk of 28-day mortality in specific subgroups of septic patients: those aged ≤55 years [HR (95% CI) 5.6164 (2.1292–14.8152)], with a lactate level of 5 mmol/L [HR (95% CI) 1.531 (1.2071–1.9418)]. In addition, a significant association was noted in patients without hypertension [HR (95% CI) 1.5788 (1.0536–2.366)] and in those without diabetes [HR (95% CI) 1.4896 (1.0719–2.0700)]. No significant interactions were detected between EPO and the incidence of various endpoints within these subgroups (Fig. 5). Furthermore, subgroup analyses were performed based on CKD status, infection type, and admission route. The results indicated no significant interactions between the subgroups, indicating that our findings are consistent and robust across different subgroups. Detailed results are provided in Appendix eTable 2.

### Mediation analyses

**Fig. 6:**
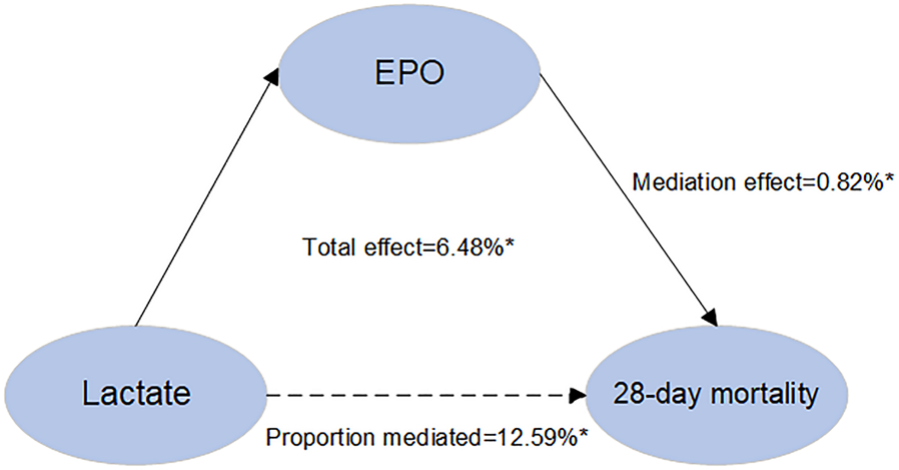
Mediated analysis model path diagram

As previously demonstrated, lactate serves as a prognostic risk factor for septic patients [29]. In this study, we established that EPO is associated with 28-day mortality and is correlated with lactate levels, prompting us to conduct mediation analyses. Causal mediation analysis was performed to investigate the potential mediating role of EPO in the relationship between lactate and 28-day mortality. Figure 6 illustrates the mediation model and pathway, with lactate designated as the independent variable, EPO as the mediator, and 28-day mortality as the dependent variable. Our findings revealed that EPO significantly mediates the association between lactate and 28-day mortality. In the unadjusted models, EPO accounted for 18.94% of the association between lactate and 28-day mortality. After adjusting for covariates, the proportion mediated through EPO was found to be 12.59% for the effect of lactate on 28-day mortality. The mediating effect of EPO on the association between lactate and 28-day mortality was significant in both the unadjusted and adjusted models (Table 5).

**Table 5:** Mediation analysis of EPO in the association between lactate and 28-day mortality

	Model 1		Model 2		Model 3	
	Coefficient (95% CI)	*P* value	Coefficient (95% CI)	*P* value	Coefficient (95% CI)	*P* value
Total effect	0.0824 (0.0578–0.1297)	<0.0001	0.0837 (0.0591–0.1302)	<0.0001	0.0648 (0.0346–0.1164)	<0.0001
Mediation effect	0.0156 (0.0073–0.3402)	<0.0001	0.0169 (0.0080–0.0256)	<0.0001	0.0082 (0.0023–0.0153)	<0.0040
Direct effect	0.0668 (0.0709–0.3152)	<0.0001	0.0668 (0.0425–0.1148)	<0.0001	0.0566 (0.0265–0.1075)	<0.0001
Proportion mediated	0.1894 (0.0827–0.3402)	<0.0001	0.2015 (0.0820–0.3249)	<0.0001	0.1259 (0.0265–0.1075)	<0.0040

Model 1: Unadjusted

Model 2: Adjusted for age, gender; hypertension, and diabetes

Model 3: Adjusted for age, gender, hypertension, diabetes, SOFA score, hemoglobin, PLT, PCT, glucose, and albumin

## Discussion

In this study, we investigated the prognostic value of EPO in sepsis patients admitted to the ICU. We found that that elevated EPO levels were significantly associated with increased 28-day and hospital mortality. Multivariate Cox regression analysis confirmed that EPO is an independent predictor of 28-day mortality, with patients in the highest EPO tertile having nearly three times the risk of death compared to those in the lowest tertile. Restricted cubic spline analysis further validated the nonlinear relationship between EPO levels and mortality risk, underscoring its potential as a prognostic marker in sepsis.

Our results are consistent with previous studies suggesting that elevated EPO levels are linked to poor outcomes in sepsis patients [23, 30, 31]. For instance, Tamion et al. [31] reported that EPO could serve as a biological marker for disease severity in critically ill patients, including those with sepsis. Similarly, Abel et al. [30] found a negative prognostic association between elevated EPO levels and outcomes in septic shock patients. However, Jiang et al. [24] reported contrasting findings, with survivors displaying higher EPO levels than non-survivors. This discrepancy may be attributed to differences in study populations, as Jiang et al. focused exclusively on non-anemic patients, whereas this study included both anemic and non-anemic patients. This highlights the importance of considering patient heterogeneity when interpreting EPO’s prognostic role. In addition, Jiang et al.’s cohort demonstrated higher lactate levels, a lower oxygenation index, and a 28-day mortality rate of 40% compared to 23% in this study, suggesting their patients had more severe clinical conditions.

Despite these findings, the mechanisms underlying the association between EPO and sepsis prognosis remain poorly understood. Preclinical studies have demonstrated that EPO possesses anti-inflammatory properties, inhibiting the release of pro-inflammatory mediators, such as tumor necrosis factor-α (TNF-α) and interleukin-1β (IL-1β) [15, 32, 33]. Furthermore, EPO treatment has been shown to improve survival in septic mice and mitigate lung injury and lethality in rat models of sepsis-associated ARDS [34, 35]. A randomized controlled trial published in the New England Journal of Medicine also reported a significant reduction in mortality among critically ill patients treated with EPO [36].

However, our findings suggest that elevated EPO levels in septic patients may be associated with poorer outcomes. We hypothesize that this discrepancy may be due to several underlying factors. First, during the early phase of sepsis, both pro-inflammatory and anti-inflammatory responses are heightened, with non-survivors exhibiting more pronounced responses than survivors [37, 38]. Elevated EPO levels in this context may reflect an intensified but dysregulated immune response. In addition, EPO elevation may represent a compensatory mechanism for hypoxia and anemia, which are common in severe infections [39]. Sepsis induces a state of systemic inflammation and widespread tissue hypoxia due to impaired perfusion, resulting in increased EPO production as the body attempts to rectify these deficits. However, it has been shown that inflammatory anemia responds poorly to EPO in sepsis, and while EPO concentrations may be elevated, they are insufficient to correct anemia and hypoxia [40]. This suggests that EPO elevation in sepsis may serve as a marker of disease severity rather than a protective response.

Notably, this study is the first to compare the predictive value of EPO with lactate, SOFA score, and inflammatory markers. We found that EPO’s predictive value for 28-day mortality is noninferior to that of the SOFA score and lactate, and superior to that of conventional inflammatory markers, such as PCT and CRP. This suggests that EPO could be a valuable addition to existing prognostic tools for sepsis.

In addition, we observed that EPO partially mediates the effect of lactate on mortality. This finding implies that EPO may contribute to the adverse outcomes associated with elevated lactate levels, providing new insights into the interplay between EPO and sepsis pathophysiology. Further research is needed to elucidate the mechanisms underlying this relationship.

In subgroup analyses, we found no significant differences in oxygenation indices across EPO level groups, nor was there a significant interaction with hypoxia levels. Similarly, sensitivity analyses and subgroup analyses based on chronic kidney disease (CKD) status yielded consistent results, indicating that the prognostic impact of EPO in sepsis is independent of hypoxia and renal function. We propose that this phenomenon may be attributed to the fact that EPO production in sepsis is influenced not only by oxygen levels and renal function but also by inflammation and anemia, both of which are potent stimulators of EPO synthesis [41–43].

Despite these significant findings, this study has several limitations. First, the single-center design may restrict the generalizability of the results, and the observational nature of the study precludes definitive conclusions regarding causality. Second, although our model accounts for numerous covariates, residual confounding cannot be entirely eliminated, which is a common issue in observational studies. Third, the relatively small size of the study population underscores the need for larger multicenter studies to validate these findings. Furthermore, a single measurement of EPO may not fully capture the dynamic changes in EPO levels during the acute phase of sepsis. Future studies should incorporate serial EPO measurements to better understand its temporal relationship with disease progression.

Future research should aim to elucidate the mechanistic pathways linking EPO to sepsis outcomes. Investigating the role of EPO in the inflammatory and immune responses during sepsis could provide deeper insights into its impact on patient prognosis. In addition, exploring therapeutic interventions targeting EPO pathways may offer new strategies for improving sepsis management and patient outcomes.

## Conclusion

Elevated EPO levels in patients with sepsis are associated with short-term prognosis and may serve as an early indicator of poor outcomes. The incorporation of EPO measurements into clinical practice could improve the risk stratification and management of sepsis patients. Further research is necessary to clarify the underlying mechanisms and explore the potential therapeutic implications of EPO in the context of sepsis.

## Supplementary Information

Additional file 1.

## Data Availability

The data sets generated and analysed during the current study are not publicly available due privacy or ethical restrictions but are available from the corresponding author on reasonable request.

## Abbreviations

EPO: Erythropoietin
PCT: Procalcitonin
CRP: C-reactive protein
WBC: White blood cell count
SIRS: Systemic inflammatory response syndrome
SOFA: Sequential organ failure assessment
aCCI: Age-adjusted charlson comorbidity index
GCS: Glasgow coma scale
ICU: Intensive care unit
ROC: Area under curve
CI: Confidence interval
TNF-α: Tumor necrosis factor-α
IL-1: Interleukin-1
IL-6: Interleukin-6
IL-8: Interleukin-8
MCV: Mean cell volume
RDW: Red cell distribution width
BUN: Blood urea nitrogen
ALT: Alanine aminotransferase
LOS ICU days: Intensive care unit length of stay
LOS hospital day: Hospital length of stay
CKD: Chronic kidney disease
