# Supplementary material for: Erythropoietin as a critical prognostic indicator in ICU patients with sepsis: a prospective observational study
Source: J Intensive Care. 2025 Mar 20;13:17. doi: 10.1186/s40560-025-00787-x (PMC11924785; doi:10.1186/s40560-025-00787-x)
Supplement: Supplementary file 1 — Additional file 1. [file 40560_2025_787_MOESM1_ESM.docx]

| **eTable 1. The association between EPO levels and 28-day mortality after excluding CKD patients.** | | | | | | |
| --- | --- | --- | --- | --- | --- | --- |
| **Categories** | **Model1** | | **Model2** | | **Model3** | |
|  | **HR (95% CI)** | ***P* -value** | **HR (95% CI)** | ***P* -value** | **HR (95% CI)** | ***P* -value** |
| **28-day mortality** | | | | | | |
| **EPO*** | 1.6866 (1.3960, 2.0376) | <0.0001 | 1.7460 (1.4412, 2.1152) | <0.0001 | 1.2961 (1.0321, 1.6277) | 0.0256 |
| **EPO tertile** | | | | | | |
| **T1** | 1.0 | | 1.0 | | 1.0 |  |
| **T2** | 1.6024 (0.6935, 3.7021) | 0.2698 | 1.6955 (0.7304, 3.9359) | 0.2192 | 1.4295 (0.6100, 3.3499) | 0.4108 |
| **T3** | 3.9376 (1.8688, 8.2967) | 0.0003 | 4.4872 (2.0676, 9.7383) | 0.0001 | 2.1797 (0.9443, 5.0312) | 0.0679 |
| **Hospital mortality** | | | | | | |
| **EPO*** | 1.3973 (1.1752, 1.6614) | 0.0001 | 1.5067 (1.2605, 1.8010) | <0.0001 | 1.0526 (0.8338, 1.3288) | 0.6664 |
| **EPO tertile** | | | | | | |
| **T1** | 1.0 |  | 1.0 |  | 1.0 |  |
| **T2** | 1.4041 (0.7501, 2.6282) | 0.2887 | 1.5288 (0.8132, 2.8744) | 0.1875 | 1.1821 (0.6137, 2.2768) | 0.6170 |
| **T3** | 2.0705 (1.1373, 3.7694) | 0.0173 | 2.5518 (1.3613, 4.7835) | 0.0035 | 1.0459 (0.4991, 2.1919) | 0.9053 |

**eTable 2. Subgroup analysis of the association between EPO and 28-day mortality**

| **Subgroup** | **N** | **HR (95%)** | | | **P value** | **P for interactor** |
| --- | --- | --- | --- | --- | --- | --- |
| **CKD** |  |  | | |  | 0.6115 |
| **no** | 232 | 1.004 (1.002,1.006) | | | 0.0001 |  |
| **yes** | 35 | 1.003 (1.001,1.006) | | | 0.0197 |  |
| **Admission route** | |  |  |  |  | 0.6989 |
| **emergency department** | 145 | 2.046 (1.439, 2.909) | | | 0.0001 |  |
| **transferred from other wards** | 122 | 1.847 (1.263, 2.701) | | | 0.0016 |  |
| **Types of infections** | |  |  |  |  | 0.1164 |
| **respiratory infections** | 176 | 1.003 (1.001, 1.005) | | | 0.0003 |  |
| **abdominal infections** | 45 | 1.006 (1.002, 1.010) | | | 0.0053 |  |
| **urinary tract infections** | 23 | 1.046 (0.976, 1.122) | | | 0.2021 |  |
| **other infections** | 23 | 1.004 (0.998, 1.011) | | | 0.1672 |  |
| **Oxygenation index** |  |  | | |  | 0.1022 |
| **<100** | 14 | 1.023 (0.987, 1.060) | | | 0.2161 |  |
| **>=100, <200** | 78 | 1.004 (1.001, 1.007) | | | 0.0083 |  |
| **>=200, <300** | 96 | 0.999 (0.993, 1.005) | | | 0.8232 |  |
| **>=300** | 79 | 1.005 (1.003, 1.008) | | | <0.0001 |  |

**eTable 3. STROBE Statement**

| **Item** | **Recommendation** |  |
| --- | --- | --- |
| **Title and abstract** | (a) Indicate the study’s design with a commonly used term in the title or the abstract | Erythropoietin as a critical prognostic indicator in ICU patients with sepsis: a prospective observational study |
|  | (b) Provide in the abstract an informative and balanced summary of what was done  and what was found | Through survival analysis, cox proportional hazards modeling, subgroup analysis, and restricted cubic spline (RCS) curves, we found that elevated EPO levels in patients with sepsis are associated with poor prognosis. |
| **Introduction** |  |  |
| Background/rationale | Explain the scientific background and rationale for the investigation being reported | The manuscript explains that sepsis is a leading cause of mortality and the need for improved biomarkers to predict outcomes. It also introduces EPO as a potential prognostic marker. The clinical relevance of circulating EPO in sepsis outcomes remains unclear, thus prompting this study to assess its prognostic value. |
| Objectives | State specific objectives, including any prespecified hypotheses | The primary objective is to assess the prognostic value of EPO levels in sepsis patients, with a focus on 28-day mortality. Elevated EPO levels are hypothesized to correlate with worse sepsis outcomes. |
| **Methods** |  |  |
| Study design | Present key elements of study design early in the paper | The study is a prospective observational study. |
| Setting | Describe the setting, locations, and relevant dates, including periods of recruitment, exposure, follow-up, and data collection | The study was conducted in the ICU of the Second Affiliated Hospital of Wenzhou Medical University, from October 1, 2022, to December 30, 2023. |
| Participants | (a) Give the eligibility criteria, and the sources and methods of selection of participants. Describe methods of follow-up | Inclusion criteria: sepsis patients meeting sepsis 3.0 criteria, and completion of laboratory tests within 48 hours of ICU admission.  Exclusion criteria: Conditions like pregnancy, malignancies, chronic illnesses (CKD stage 4 and 5), or those receiving exogenous EPO therapy.  Follow-up: follow-up was done for 28 days to assess mortality. |
|  | (b) For matched studies, give matching criteria and number of exposed and unexposed | None |
| Variables | Clearly define all outcomes, exposures, predictors, potential confounders, and effect modifiers. Give diagnostic criteria, if applicable | Outcomes: 28-day mortality (primary outcome) and hospital mortality (secondary outcome).  Exposures: EPO levels, lactate, SOFA score, etc.  Confounders: age, gender, comorbidities like diabetes and hypertension.  Diagnostic criteria: Sepsis3.0 |
| Data sources/ measurement | For each variable of interest, give sources of data and details of methods of assessment (measurement). Describe comparability of assessment methods if there is more than one group | Data was collected from medical records and laboratory results. The methods of assessment are provided in the methods section (e.g., EPO, lactate, SOFA score). |
| Bias | Describe any efforts to address potential sources of bias | Efforts to reduce bias include controlling for confounders such as age, comorbidities, and using Cox regression models. |
| Study size | Explain how the study size was arrived at | Based on previous literature |
| Quantitative variables | Explain how quantitative variables were handled in the analyses. If applicable, describe which groupings were chosen and why | The normality of continuous variables was assessed via the Kolmogorov‒Smirnov (K‒S) test. Categorical variables are expressed as numbers and percentages (%), with comparisons performed via the chi-square test or Fisher's exact test as appropriate. According to the 28-day mortality and grouping by the tertiles of EPO concentrations |
| Statistical methods | (a) Describe all statistical methods, including those used to control for confounding | Statistical methods include Cox regression, Kaplan-Meier survival analysis, and restricted cubic splines for nonlinear relationships, subgroup analysis and sensitivity analysis. Adjusting for confounding factors in the Cox proportional hazards model. |
|  | (b) Describe any methods used to examine subgroups and interactions | Subgroup analyses were conducted based on variables like age, gender, SOFA score, oxygenation index, CKD, hypertension, diabetes and infection types. |
|  | (c) Explain how missing data were addressed | No missing data. |
|  | (d) If applicable, explain how loss to follow-up was addressed | Not applicable |
|  | (e) Describe any sensitivity analyses | Sensitivity analysis excluded CKD patients |
| **Results** |  |  |
| Participants | (a) Report numbers of individuals at each stage of study—eg numbers potentially eligible, examined for eligibility, confirmed eligible, included in the study, completing follow-up, and analysed | Numbers at each stage: 267 patients included, with 83 excluded due to exclusion criteria. |
|  | (b) Give reasons for non-participation at each stage | 1.Age less than 18 years (n=4)  2.Receiving exogenous EPO therapy (n=43)  3.Pregnancy or lactation (n=3)  4.Immunosuppressed State (n=17)  5.Chronic kidney disease stage 4 and stage 5 (n=10)  6.Interhospital transfer cases (n=6) |
|  | (c) Consider use of a flow diagram | A flowchart of patient selection is included (Figure 1). |
| Descriptive data | (a) Give characteristics of study participants (eg demographic, clinical, social) and information on exposures and potential confounders | The baseline characteristics of the participants are provided in Table 1, which includes demographics and clinical features. |
|  | (b) Indicate number of participants with missing data for each variable of interest | No missing data. |
|  | (c) Summarise follow-up time (eg, average and total amount) | Minimum follow-up time: 28day |
| Outcome data | Report numbers of outcome events or summary measures over time | Mortality outcomes are presented for both 28-day and hospital mortality. |
| Main results | (a) Give unadjusted estimates and, if applicable, confounder-adjusted estimates and their precision (eg, 95% confidence interval). Make clear which confounders were adjusted for and why they were included | The unadjusted estimate was 1.7169 (95% CI: 1.4529, 2.0288), while the confounder-adjusted estimate was 1.3086 (95% CI: 1.0304, 1.6618). The manuscript clearly specifies the adjusted variables. |
|  | (b) Report category boundaries when continuous variables were categorized | The mean (Q1–Q3) EPO concentrations for each group were 29.23 (12.64–93.60), 22.81 (11.29–51.66), and 75.25 (30.10–447.19), respectively. |
|  | (c) If relevant, consider translating estimates of relative risk into absolute risk for a meaningful time period | None |
| Other analyses | Report other analyses done—eg analyses of subgroups and interactions, and sensitivity analyses | Subgroup analysis and causal mediation analysis are included. Sensitivity analysis was conducted by excluding CKD patients. |
| **Discussion** |  |  |
| Key results | Summarise key results with reference to study objectives | Higher EPO levels are significantly associated with increased mortality in sepsis patients. |
| Limitations | Discuss limitations of the study, taking into account sources of potential bias or imprecision. Discuss both direction and magnitude of any potential bias | Discusses limitations such as single-center design, residual confounding, and potential biases. |
| Interpretation | Give a cautious overall interpretation of results considering objectives, limitations, multiplicity of analyses, results from similar studies, and other relevant evidence | The study suggests that elevated EPO may act as a prognostic marker for sepsis, but the exact mechanism remains unclear. |
| Generalisability | Discuss the generalisability (external validity) of the study results | The findings are limited by the single-center design and require further validation in multicenter studies. |
| **Other information** |  |  |
| Funding | Give the source of funding and the role of the funders for the present study and, if applicable, for the original study on which the present article is based | This work was supported by grants from the National Natural Science Foundation of China (82002093), Natural Science Foundation of Zhejiang Province (Y24H150028), National Key RD Program of China (2022YFC2504404), Wenzhou Municipal Science and Technology Bureau (Y20220086), Clinical Research Foundation of the 2nd Affiliated Hospital of Wenzhou Medical University (SAHoWMU-CR2018-11-134). |

**eTable 4. Summary of disease distribution and quantities**

| **Disease** | **Quantity** |
| --- | --- |
| **Heart disease** | 40 |
| Atrial fibrillation | 16 |
| First-degree atrioventricular block | 1 |
| Coronary artery disease | 10 |
| Purulent pericarditis | 1 |
| Dilated cardiomyopathy | 1 |
| Pericardial effusion | 2 |
| Heart failure | 9 |
| **Lung disease** | 18 |
| COPD | 3 |
| Pulmonary embolism | 1 |
| Structural lung disease | 1 |
| Bronchiectasis | 2 |
| Asthma | 1 |
| Severe pneumonia | 10 |
| **Liver disease** | 34 |
| Hepatitis C | 2 |
| Hepatitis B | 9 |
| Liver cyst | 1 |
| Intrahepatic biliary stones | 1 |
| Liver cirrhosis | 4 |
| Acute liver failure | 14 |
| Fatty liver disease | 3 |
| **Malignant tumor** | 50 |
| Nasopharyngeal cancer | 1 |
| Bile duct cancer | 3 |
| Lung cancer | 13 |
| Liver cancer | 9 |
| Malignant thyroid tumor | 1 |
| Descending colon tumor | 2 |
| Brain tumor | 1 |
| Prostate cancer | 3 |
| Malignant tongue tumor | 1 |
| Kidney cancer | 1 |
| Adrenal tumor | 1 |
| Esophageal cancer | 4 |
| Ureteral tumor | 2 |
| Stomach cancer | 1 |
| Pancreatic cancer | 1 |
| Rectal cancer | 5 |
